# Beneficial effects of cherry consumption as a dietary intervention for metabolic, hepatic and vascular complications in type 2 diabetic rats

**DOI:** 10.1186/s12933-018-0744-6

**Published:** 2018-07-20

**Authors:** Remmelt Van der Werf, Catherine Walter, William Bietiger, Elodie Seyfritz, Carole Mura, Claude Peronet, Julie Legrandois, Dalal Werner, Said Ennahar, Fabien Digel, Elisa Maillard-Pedracini, Michel Pinget, Nathalie Jeandidier, Eric Marchioni, Séverine Sigrist, Stéphanie Dal

**Affiliations:** 1 DIATHEC EA 7294, Fédération de Médecine Translationnelle de Strasbourg, Centre Européen d’Etude du Diabète, Boulevard René Leriche, Université de Strasbourg, 67000 Strasbourg, France; 2 Aérial, Illkirch, France; 3 IPHC-LC4, UMR 7178, Faculté de Pharmacie, Equipe de Chimie Analytique des Molécules BioActives, Illkirch, France; 4 Interprofession des Fruits et Légumes d’Alsace (IFLA), Sainte Croix en Plaine, France; 5 Structure d’Endocrinologie, Diabète, Nutrition et Addictologie, Pôle NUDE, Hôpitaux Universitaires de Strasbourg, (HUS), 67000 Strasbourg, France

**Keywords:** Hepatic diabetic-complications, Endothelial dysfunction, Oxidative stress, Nutritional antioxidant approach, Cherry, Diabetes prevention

## Abstract

**Background:**

Oxidative stress (OS) plays an important role in type 2 diabetes (T2D) pathogenesis and its complications. New therapies target natural antioxidants as an alternative and/or supplemental strategy to prevent and control them. Our previous chemical and biological studies highlighted the important antioxidant activities of cherries, among other fruits and vegetables, thus we aimed to determine in vivo effects of 2-month long cherry consumption using a high-fat/high-fructose (HFHF) model of diabetic-rats (Lozano et al. in Nutr Metab 13:15, 2016).

**Methods:**

After 2 months of HFHF, male Wistar rats were divided into: HFHF and HFHF enriched in cherry (nutritional approach) or standard diet ND (lifestyle measures) and ND plus cherry during 2 months. Metabolic, lipidic, oxidative parameters were quantified. Tissues (liver, pancreas and vessels) OS were assessed and hepatic (steatosis, fibrosis, inflammation) and vascular (endothelial dysfunction) complications were characterized.

**Results:**

T2D was induced after 2 months of HFHF diet, characterized by systemic hyperglycaemia, hyperinsulinemia, glucose intolerance, dyslipidaemia, hyperleptinemia, and oxidative stress associated with endothelial dysfunction and hepatic complications. Cherry consumption for 2 months, in addition to lifestyle measures, in T2D-rats decreased and normalized the systemic disturbances, including oxidative stress complications. Moreover, in the vessel, cherry consumption decreased oxidative stress and increased endothelial nitric oxide (NO) synthase levels, thus increasing NO bioavailability, ensuring vascular homeostasis. In the liver, cherry consumption decreased oxidative stress by inhibiting NADPH oxidase subunit p22phox expression, nuclear factor erythroid-2 related factor 2 (Nrf2) degradation and the formation of reactive oxygen species. It inhibited the activation of sterol regulatory element-binding proteins (1c and 2) and carbohydrate-responsive element-binding protein, and thus decreased steatosis as observed in T2D rats. This led to the improvement of metabolic profiles, together with endothelial and hepatic function improvements.

**Conclusion:**

Cherry consumption normalized vascular function and controlled hepatic complications, thus reduced the risk of diabetic metabolic disorders. These results demonstrate that a nutritional intervention with a focus on OS could prevent and/or delay the onset of vascular and hepatic complications related to T2D.

**Electronic supplementary material:**

The online version of this article (10.1186/s12933-018-0744-6) contains supplementary material, which is available to authorized users.

## Background

Oxidative stress is widely accepted to be involved in the pathogenesis of type 2 diabetes (T2D) and its complications [2]. Oxidative stress occurs because of an imbalance between antioxidants (enzymes, vitamins, and proteins) and pro-oxidants (UV radiation, alcohol, and smoking) [3] leading to a bipolar process involving the generation of reactive oxygen species (ROS) and a decrease in plasma antioxidants. Many disorders observed in T2D patients such as hyperinsulinemia [4], hyperlipidaemia [5], glucose fluctuations [6, 7], hyperglycaemia [8], and inflammation [9–11], induce formation of ROS and exacerbate oxidative stress [11, 12]. Moreover, we have recently demonstrated in T2D rat models that oxidative stress is involved in both hepatic and vascular complications [1]. In fact, in T2D, the liver is involved in the accumulation of triglycerides, development of hepatic insulin resistance, and development of non-alcoholic steatohepatitis (NASH) [1, 13]. The liver plays a major role in the regulation of blood glucose levels in close cooperation with the pancreas and other peripheral tissues; however, several studies have reported an association between non-alcoholic fatty liver disease (NAFLD) and cardiovascular disease-related complications [14]. Vessels, and more precisely the internal layer endothelium, are the first sites for the development of complications such as high cholesterol and high blood pressure [15], obesity and visceral fat distribution [16], impaired fasting glucose and hyperglycaemia [17] and, more recently hypoglycaemia [18] and insulin resistance [19]. Under these pathological conditions, the strategic equilibrium between relaxant and contractor factors is lost in favour of pro-mitogenic, pro-aggregation mediators and inflammation, leading to endothelial dysfunction as observed in T2D patients [11, 20]. Diabetic vascular complications also lead to further functional deterioration inducing coronary arteriosclerosis, neuropathy, nephropathy… [21], and are associated with cardiovascular and all-cause mortality in patients with diabetes [22].

Lifestyle modifications/changes are the first essential pillar of the management of patients with diabetes, even before the introduction of a drug treatment. Lifestyle modifications prevent significant changes in blood glucose levels, decrease insulin resistance, and promote weight loss in order to limit the development of diabetic complications and attenuate its severity [23]. In addition to nutritional benefit, fruits, vegetables, cereals and beverages supplies bioactive molecules (such as vitamins and polyphenols) possessing antioxidant properties, providing a real advantage in the prevention of chronic diseases, such as obesity, diabetes, cardiovascular diseases and cancer. In fact, some studies have revealed an inverse relationship between the risk of cardiovascular mortality or morbidity linked to T2D and the consumption of polyphenol-rich products (e.g. red wine, cocoa, and tea) [24–26]. In 2017, a large epidemiological study in Chinese adults found that an increased consumption of fresh fruits was associated with a significantly lower risk of diabetes and, among diabetic individuals, lower risks of death and development of major vascular complications [27]. The consumption of fresh fruits that contain several polyphenols and vitamins can increase antioxidant levels, in addition to their direct effects on blood vessels and, in particular, on the endothelium [28]. High consumption of fruits and vegetables has been associated with a decrease in the incidence of chronic diseases and complications, including obesity and diabetes [29, 30], and these beneficial effects have been attributed to phytochemicals.

Polyphenolic substances have received widespread attention because of their interesting biological activities, bioavailability and protective role against oxidative stress and free radical damage [31]. Our recent work has demonstrated the beneficial impact of polyphenol consumption (red wine) in prevention of metabolic syndrome complications in vivo [32] and in the protection of β-cells from loss of viability induced by oxidative stress in vitro (red wine and green tea) [33]. Recently, there has been a considerable interest in identifying natural polyphenols from plants, fruits, and vegetables that play an important role in the management of disorders involving oxidative stress, such as diabetes and its complications [29, 30]. Our recent work on fruits and vegetables has shown, using a new high performance liquid chromatography (HPLC) method coupled with a post-column reaction system relaying 2,2′-azino-bis-(3-ethylbenzthiazoline-6-sulfonic acid) (ABTS^.−^) bleaching assay [34], the ability of some fruits and vegetables to scavenge ROS. Moreover, the complement of these chemical studies by tests carried out on β-cells using the fluorescent probe DCFH-DA demonstrated their in vitro antioxidant capacity and identified the most active fruits and vegetables. Notably, cherries were identified as an active scavenging fruit with a high level of polyphenols [35].

Cherries (*Rosaceae*) are considered a nutrient dense food with a relatively low caloric content and a significant amount of important nutrients and bioactive food components [36]. Cherries are one of the richest sources of anthocyanins and antioxidants-substances and are more effective than vitamin C and are four times more potent than vitamin E in antioxidant activity [37]. The anthocyanins in cherries give a dark red colour [38] and have been shown to be associated with the prevention of lifestyle-related diseases such as cancer, diabetes and cardiovascular diseases [39] and neurodegenerative disease [40]. Moreover, recently, Keane et al. [41] demonstrated that the acute supplementation with tart cherry juice can lower blood pressure and improve some aspects of exercise performance, highlighting the beneficial impact of bioactive compound and physical activity. However, there is little data available on the use of cherries to reduce or prevent diabetes and its complications. Our previous study demonstrated that Regina cherries containing several phenolic compounds, including anthocyanins and flavones [35], demonstrated high antioxidant activities, with the new HPLC-ABTS^−^ bleaching assay [34]. In fact, Regina cherry (*Prunus avium*) is known as sweet cherry and considered nutrient dense food with a relatively low caloric content and a significant amount of important nutrients [42] and bioactive food. Regina Cherry has twice higher chemical radical scavenging activities than Folfer cherry with an IC50 lower than 35 mg of fresh matter/mL in comparison to higher than 160 mg of fresh matter/mL for Folfer cherry [34, 35]. Moreover, a study reported that cherry consumption increased plasma lipophilic antioxidant capacity [43], which is severely decreased in patients with diabetes [44].

Despite widely available antidiabetic medicines in the pharmaceutical market, diabetes and its related complications continue to be a major medical problem. Due to a low level of expression of antioxidant enzymes in the pancreas of patients with diabetes [45], combinations of conventional antidiabetic treatments with antioxidants were prioritized [46]. The central role of oxidative stress in the pathophysiology of T2D and its complications is now well demonstrated and some studies support the protective effects of various polyphenol-rich foods against chronic diseases. However, based on a selection of antioxidant capacity fruits and vegetables, a robust demonstration on the mechanism of action of polyphenols extract on diabetes and its complications has to be performed. The aim of this study was then to demonstrate the effect of long-term cherry consumption in a T2D model with endothelial dysfunction and non-alcoholic fatty liver disease (NAFLD) complications. We determined the effect of 2 months of cherry consumption added to a high fat high fructose (HFHF) diet or a normal diet (ND) through two strategies: nutraceutical or lifestyle interventions. We focused on the effects of these two treatments on metabolic, oxidative, and inflammatory parameters and vascular, pancreatic, and hepatic functions.

## Methods

### Experimental protocols

#### Ethics statement

The study was performed in accordance with the “Guide for the Care and Use of Laboratory Animals” published by the US National Institutes of Health (NIH publication No. 85-23, revised 1996), and the present protocol was approved by the local ethics committee (Comité Régional d’Ethique en Matière d’Expérimentation Animale CREMEAS, approval AL/65/72/02/13). All efforts were made to minimize animal suffering and minimize the number of animals used.

#### Animal and diet compositions

Forty-eight male Wistar rats (7 weeks old; 246 ± 4.8 g), supplied by Depré (Saint Doulchard, France), were housed in a temperature-controlled room, in a 12 h light/dark cycle environment with ad libitum access to water and food throughout the study. After 1 week of acclimation and quarantine (299 ± 5.1 g), T2D was induced with the addition of the high fat diet “WESTERN RD” from SDS (Special Diets Services, Saint Gratien, France) added to 25% fructose in water as beverage [1]. This HFHF diet was compared to a ND from SAFE (Augy, France). Cherries (*Var. Regina*) from IFLA (Interprofession des Fruits et Légumes d’Alsace, France) were lyophilized (CEVA, Centre d’Etude et de Valorisation des Algues, Pleubian, France), crushed (Technopoudre, Ancenis, France) and then incorporated in both foods at 10% concentration according to the protocol outlined below. Food compositions are presented in Additional file 1: Table S1.

#### Course of study

After 2 months, HFHF rats (547 ± 5.0 g; 1.31 ± 0.02 g/L of fasting glucose) were randomly divided into four groups. The first two groups, with access to a HFHF diet with or without cherry enrichment (respectively, HFHFCherry or HFHF), represented a ‘nutraceutical approach’. The second two groups were shifted to ND (HFHF/ND) or ND with cherry enrichment (HFHF/NDCherry) and represented ‘dietary lifestyle measures’. The groups were compared to ND rats (494.5 ± 10.0 g; 0.97 ± 0.03 g/L of fasting glucose) which received only ND for 2 more months. The body weight and calorie intake of each animal was recorded once a week. Body weight, as well as abdominal circumference were measured to calculate the body mass index. Capillary glucose levels were measured and tail vein blood samples were taken to estimate metabolic parameters. After anaesthesia with an intraperitoneal injection of 50 mg/kg pentobarbital (Centravet, France), blood was drawn from the abdominal aorta, plasma and serum were frozen in liquid nitrogen and stored at − 80 °C after centrifugation (4 °C, 2 min, 10,000×*g*) for later biochemical analysis. Liver and abdominal fat were weighed. The liver, pancreas and mesenteric artery were cleaned and embedded in Tissue-Tek^®^ OCT (Optimal Cutting Temperature compound, Leica Microsystem SAS, Nanterre, France) and directly frozen in liquid nitrogen and stored at − 80 °C. Six rats were sacrificed at the beginning of the study (control), six ND rats and six HFHF rats after 2 months of diet and six rats from all groups after 4 months (2 months of specific diets).

### Biochemical plasmatic analysis

#### Plasmatic metabolic parameters

Fasting blood glucose was determined in plasma (glucose RTU^®^, Biomérieux, France) and glucose tolerance was evaluated based on intraperitoneal glucose tolerance (IpGTT) of fasting rats. Capillary glycaemia at baseline and 15, 30, 60, and 120 min post intraperitoneal (IP)-injection of 2 g/kg glucose (20% solution) was measured with a glucometer (Accu-Chek Performa^®^, Roche Diagnostic, France). Blood samples were collected from the tail vein at 0 and 60 min post injection, in order to measure blood glucose and C-peptide levels (Elisa C-peptide kit, Mercodia, Uppsala, Sweden) to evaluate insulin sensitivity. Measuring C-peptide was preferred to measuring insulin for evaluating insulinemia because it is more stable in blood and is not affected by haemolysis [47]. Insulin resistance (IR) was evaluated using the homeostasis model assessment (HOMA2). HOMA2-IR was calculated for fasting plasma glucose and fasting C-peptide using the HOMA2 model calculator (http://www.dtu.ox.ac.uk/homa). Fasting leptin was measured by ELISA (Elisa Leptin kit, Linco Research Inc., St. Louis, MO, USA) as an index of fat mass [48], triglycerides (TG) (Triglyceride Quantification Kit, Abcam, Paris, France) and free fatty acids (FFA) (Free Fatty Acid Quantification Kit, Abcam) were measured by ELISA. Plasma cholesterol (Chol) was measured using the Cholesterol RTU™ (Biomérieux, Lyon, France) colorimetric method and a cholesterol calibrator. All parameters were measured once a month.

#### Plasmatic oxidative parameters

Plasmatic lipid peroxidation as a consequence of oxidative stress was estimated by measuring thiobarbituric acid reactive substances (TBARS) using a kit (OxiSelect™ TBARS Assay Kit-MDA Quantitation, Cell Biolabs Inc., San Diego, CA, USA) according to the manufacturer’s instructions, and expressed in µmol/L malondialdehyde (MDA). Superoxide dismutase (SOD) and catalase activities were measured according to the manufacturer’s instructions (Superoxide dismutase assay kit and Catalase Assay Kit, Abcam, Paris, France) and expressed, respectively, in percent of inhibition rate and μmol/L. Total antioxidant capacity (TAOC) with the radical cation ABTS^•+^ (2,2′-azino-bis-(3)-ethylbenzthioazoline-6-sulfonic acid, VWR, Fontenay sous Bois, France) was determined using 6-hydroxy-2, 5, 7, 8-tetramethylchromane-2-carboxylic acid (Trolox; Sigma-Aldrich, St Quentin Fallavier, France) equivalent, as previously described for plasma [1].

### Histological and functional hepatic and pancreatic studies

#### Morphological analysis

The degree of hepatic histological changes was assessed on 10-µm cryosections fixed with 4% paraformaldehyde by eosin/hematoxylin coloration and Oil Red O staining (steatosis). Steatosis was evaluated according to the standard Kleiner Classification [49] of grading and staging. Degree of steatosis was scored as the percentage of hepatocytes per lipid droplet: 0 (less than 5%), 1 (from 5 to 33%), 2 (from 33 to 66%) and 3 (higher than 66%).

In situ hepatic inflammation was determined as previously described [50] on 10 µm-cryosections fixed and incubated with rabbit anti-Iba-1 (Rat, 1:1000, Wako Chemicals GmbH, Germany). Macrophage density corresponded to the percentage of brown pixels per field and was expressed as a percentage of area. Six slides were prepared for each animal and five fields were analysed per slide at a magnification of 20×.

Hepatic and pancreatic oxidative stress was performed with a dihydroethidine (DHE) probe as described above according to a previous study [1]. Unfixed 10 µm-thick sections were treated with DHE (2.5 µM) and incubated in a light-protected humidified chamber at 37 °C for 30 min. The level of ROS was determined using microscopy and whole fluorescence of tissue was quantified with the microscope assistant (NIS-Elements BR, Nikon, France) and expressed as a percentage of that in ND rats.

#### Functional analysis

Extraction and quantification of triglyceride (Abcam) and cholesterol (Cholesterol RTU™, Biomérieux) were performed on a piece of fresh liver (100 mg) according to the manufacturer’s instructions. Extraction and quantification of glycogen content were also performed on a piece of fresh liver (100 mg) according to the manufacturer’s instructions and as previously described [1] and expressed as glycogen/mg of liver.

#### Western blotting

Total protein (80 mg) was separated on a 4–12% Bis–Tris CriterionTM XT Precast Gel (Bio-Rad, Marne-La-Coquette, France) and transferred to an Immobilon polyvinylidene difluoride (PVDF) membrane (Millipore, Molsheim, France). Antibodies against -ChREBP (1:1000, rabbit) from Novus Biologicals Canada (Oakville, Canada), -SREBP1 (1/200, mouse) and -SREBP2 (1/500, rabbit) from abcam, -p22phox (1/500, goat) and –Nrf2 (1/1000) from Santa Cruz (Dallas, TX, USA) were incubated with membranes overnight at 4 °C. Membranes were incubated for 1 h at room temperature with a corresponding horseradish peroxidase (HRP)-conjugated secondary antibody (1/2000, Sigma-Aldrich) and developed using the Luminata™ Forte Western HRP substrate (Millipore, Molsheim, France) with Chemidoc XRS (Bio-Rad, Marne-La-Coquette, France). The relative quantity of the protein of interest compared with the reference protein β-actin (1/500, mouse) from Santa Cruz or GAPDH (1/500, rabbit) from Ozyme (Saint Quentin, France) was measured with Image J software (NIH, USA).

### Histological and functional vascular studies

#### Vascular reactivity studies

The main superior mesenteric artery rings were suspended in organ baths to determine changes in isometric tension, as described previously [51]. The nitric oxide (NO)-mediated component of relaxation was determined in the presence of indomethacin (10 μM) and charybdotoxin plus apamin (100 nM each) to rule out the formation of vasoactive prostanoids and the endothelium-derived hyperpolarizing factor (EDHF), respectively. Rings were pre-contracted with phenylephrine (PE) (1 µmol/L) before the construction of a concentration-relaxation curve respective to acetylcholine (Ach) (0.1–10 µmol/L) to test endothelial calcium-dependent relaxation [52]. Relaxations were expressed as percentage of the reversal of the contraction induced by PE.

#### In situ mesenteric oxidative stress and immunochemical characterization

The oxidative fluorescent dye dihydroethidine (DHE) was used to evaluate in situ formation of ROS as describe above. Endothelial NO synthase (eNOS, 1/100, BD Biosciences) and 3-nitrotyrosine (1/100, Millipore, Molsheim, France) expression and localization were determined on 10 µm-cryosections of mesenteric arteries, fixed with 4% paraformaldehyde and incubated with both antibodies. The corresponding anti-mouse IgGs coupled to Alexa 488 (1/200, Invitrogen, Molecular Probes) were used as secondary antibodies. Fluorescence was determined using microscopy, quantified with the microscope assistant (NIS-Elements BR, Nikon, France) and expressed as a percentage of that in ND rats.

### Statistical analysis

Values are expressed as mean ± SEM, and n indicates the number of rats. Statistical analysis was performed with Student’s *t* test for unpaired data or ANOVA followed by LSD test after normality test validation protected least-significant difference tests, where appropriate (Statistica^®^ version 12, StatSoft, France). If normality was violated, we used log transform. P < 0.05 was considered to be statistically significant.

## Results

### Dietary intervention improved metabolic control of HFHF rats

**Fig. 1:**
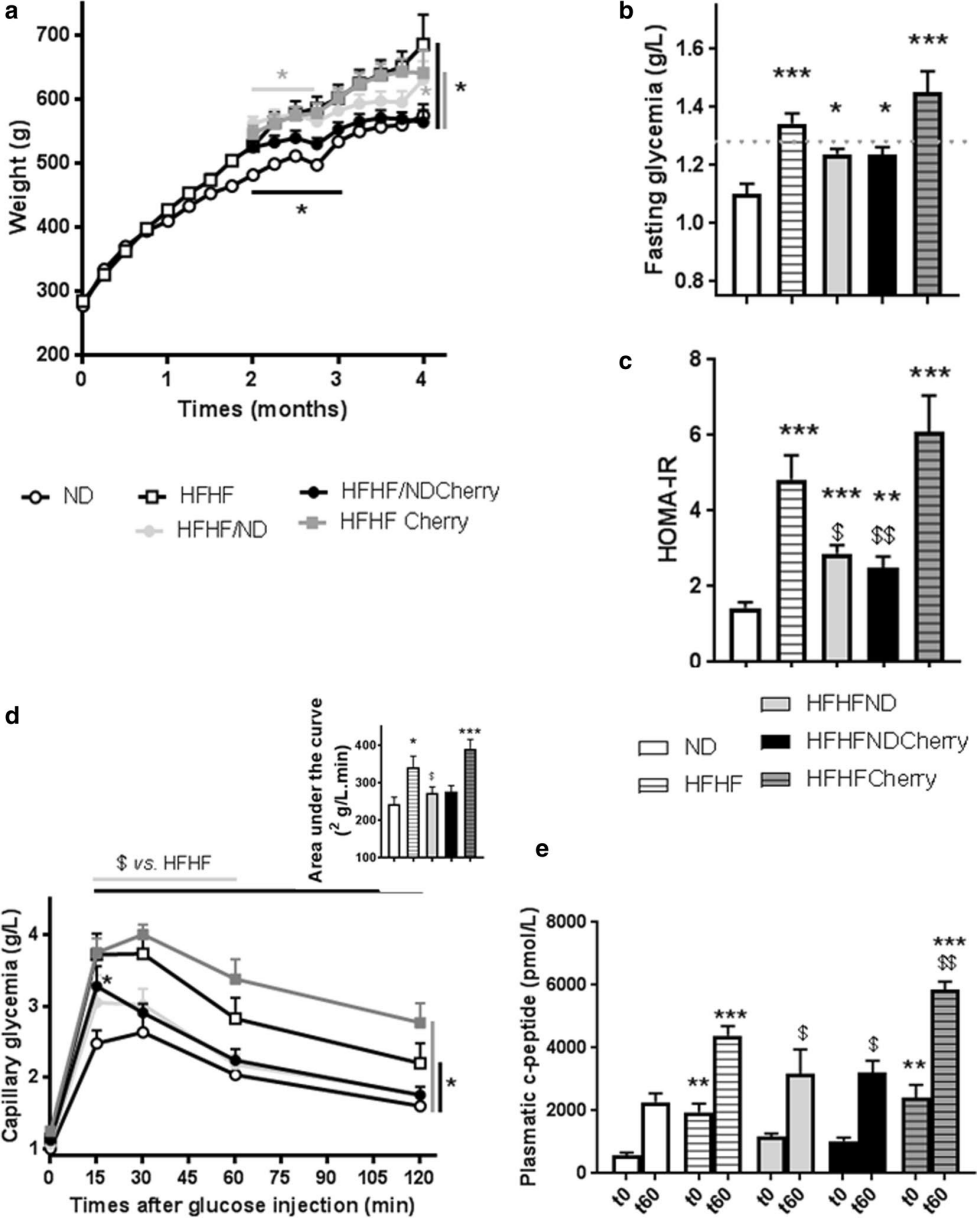
Impact of cherry consumption on metabolic characteristics of rats 4 months into the experimental period. Physiological and plasmatic metabolic impacts of cherry consumption on **a** weight gain evolution, **b** fasting glycaemia, **c** HOMA-IR, **d** capillary glycaemia and area under the curve during ipGTT, **e** fasting plasmatic c-peptid (t0) and 60 min (t60) after glucose injection during ipGTT. Results represent the mean of 6 different experiments ± SEM after 4 months of normal diet (ND), high fat high fructose (HFHF) diet, HFHF 2 months + ND 2 months (HFHF/ND); HFHF 2 months + ND with cherry supplementation 2 months (HFHF/NDCherry) and HFHF 2 months + HFHF with cherry supplementation 2 months groups (HFHFCherry). Asterisk represents significant results vs. ND; dollar vs. HFHF

**Fig. 2:**
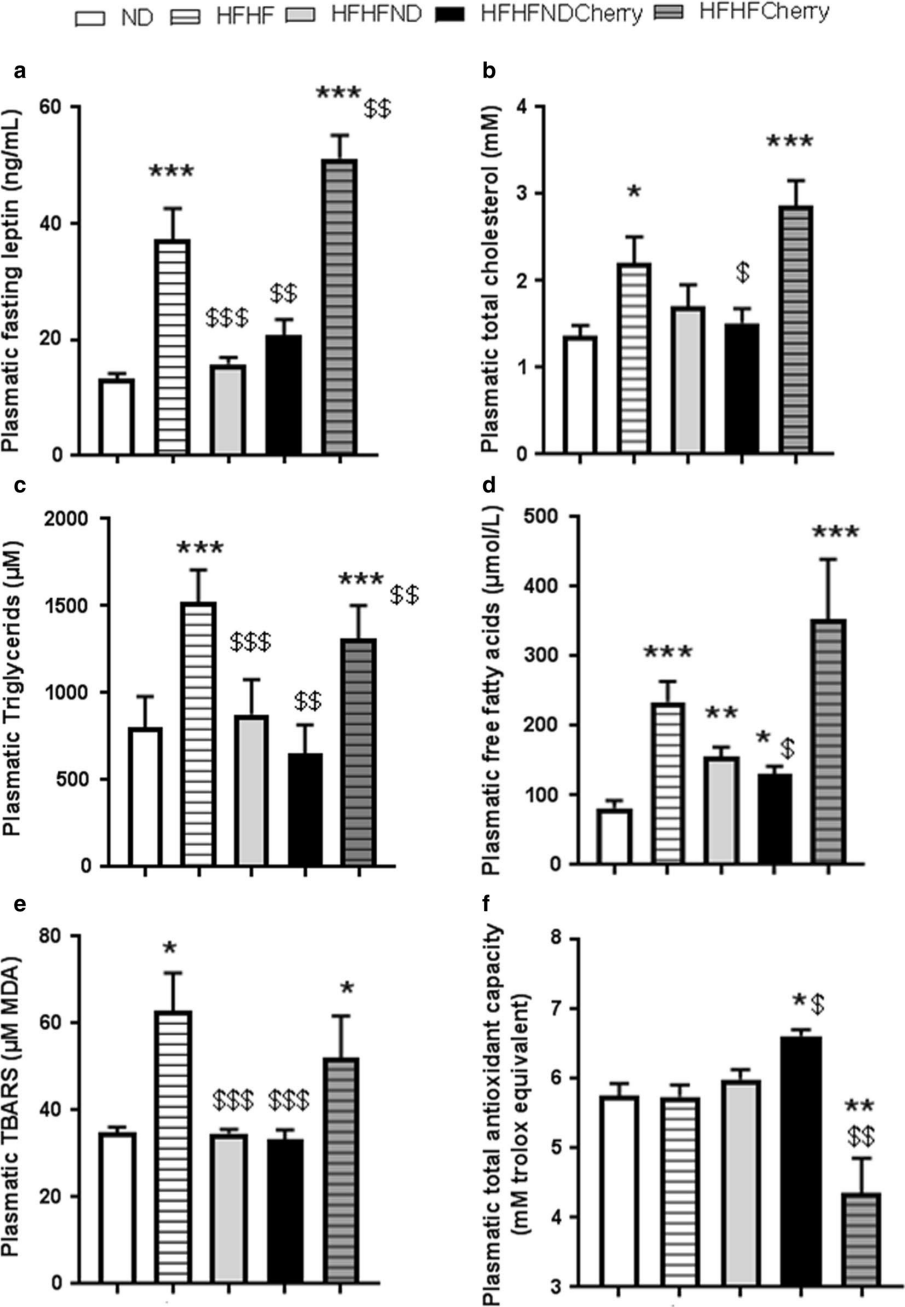
Impact of cherry consumption on plasmatic lipidic profiles, oxidative complications and hepatic glycogen characteristics of rats 4 months into the experimental period. Impacts of cherry consumption on **a** fasting leptin, **b** total cholesterol, **c** triglycerides, **d** free fatty acids, **e** TBARS formation and *F)* total antioxidant capacity. Results represent the mean of 6 different experiments ± SEM after 4 months of normal diet (ND), high fat high fructose (HFHF) diet, HFHF 2 months + ND 2 months (HFHF/ND), HFHF 2 months + ND with cherry supplementation 2 months (HFHF/NDCherry) and HFHF 2 months + HFHF with cherry supplementation 2 months groups (HFHFCherry). Asterisk represents significant results vs. ND; dollar vs. HFHF

HFHF rats maintained a significantly higher weight gain, body mass index and body weight (Table 1, Fig. 1a) than ND rats until the end of the study period. This increase was associated after 4 months with higher abdominal circumference, abdominal fat (Table 1) and hyperleptinemia (37.2 ± 5.4 vs. 13.2 ± 0.9 ng/mL) (Fig. 2a). Moreover, HFHF rats developed fasting glycaemia (defined as glucose levels > 1.26 g/L; 1.35 ± 0.04 vs. 1.10 ± 0.04 g/L) and insulin resistance (defined as HOMA-IR value > 2.4; 4.84 ± 0.63 vs. 1.43 ± 0.15) while ND rates did not (Fig. 1b, c). Further, HFHF rats developed glucose intolerance with area under the curve (343 ± 29 vs. 244 ± 18) and C-peptidemia higher than in ND rats (during ipGTT: *t0′*: 1954 ± 267 vs. 583 ± 81; *t60′*: 4398 ± 299 vs. 2274 ± 284 pmol/L) at a faster rate than the ND rats (Fig. 1d, e). Finally, higher cholesterol (2.2 ± 0.3 vs. 1.4 ± 0.1 mM), triglyceride (1523 ± 185 vs. 805 ± 175 µM) and free fatty acid (234 ± 29.4 vs. 82 ± 10.6 µM) levels revealed that dyslipidaemia was induced by 4 months of HFHF diet administration (Fig. 2b–d).

**Table 1:** Characteristics of rats after 4 months of experimental period

Variables	ND	HFHF	HFHF/ND	HFHF/NDCherry	HFHFCherry
*Physiological variables*					
Weight gain from 2 to 4 months (g)	63.4 ± 6.9	97.5 ± 4.60*	13 ± 12.14***^$$$^	36.12 ± 9.5^$$$^	95.12 ± 12.0
Final body weight (g)	558 ± 15.3	628 ± 12.6**	570 ± 13.4^$^	581 ± 12.2^$^	652.95 ± 20.71***
Body mass index (BMI) (g/cm^2^)	0.78 ± 0.003	0.86 ± 0.02*	0.76 ± 0.02^$^	0.79 ± 0.03	0.88 ± 0.05*
Abdominal circumference (cm)	22.14 ± 0.59	24.9 ± 0.78*	22.08 ± 1.34^$^	23.5 ± 0.62	25.25 ± 0.98*
Abdominal fat (g) (% vs. total weight)	12.22 ± 1.74	34.60 ± 1.38***	18.33 ± 3.39^$$$^	17.92 ± 2.43^$$$^	29.85 ± 3.57***
	2.17 ± 0.27	4.37 ± 0.96***	3.17 ± 0.49^$$$^	3.07 ± 0.40^$$$^	4.58 ± 0.52***
*Plasmatic lipidic profile*					
Fasting leptin (ng/mL)	13.23 ± 0.99	37.24 ± 5.39***	15.88 ± 1.08^$$$^	20.77 ± 13.87^$$^	51.26 ± 3.96***^$$^
Total cholesterol (mM)	1.37 ± 0.12	2.21 ± 0.30**	1.71 ± 0.25	1.52 ± 0.16^$^	2.86 ± 0.29***
Triglycerides (μM)	805.2 ± 175.2	1523 ± 184.6**	878 ± 199.7^$^	650.2 ± 164.7^$$^	1313 ± 189.4***^$$$^
Free fatty acids (μmol/L)	82.11 ± 10.64	234.4 ± 29.39***	155.3 ± 14.12*^$$^	130 ± 11.67*^$^	354 ± 85.71***
*Plasmatic oxidative profile*					
Lipid peroxide, TBARS (μM malondialdehyde)	35 ± 1.14	62.81 ± 8.86*	34.7 ± 0.91^$$$^	33.41 ± 2.08^$$$^	52.2 ± 9.54*
Total antioxidante capacity (mM trolox equivalent)	5.75 ± 0.18	5.75 ± 1.167	5.99 ± 0.131	6.62 ± 0.086*^$^	4.36 ± 0.492**^$$^

Experiments were performed six times

* Significant difference vs. ND; $ vs. HFHF; * and $ : P < 0.05; ** and $$ : P < 0.01; *** and $$$ : P < 0.001

The HFHFCherry diet did not have a beneficial impact on metabolic and lipidic parameters (Figs. 1, 2a–d). In fact, HFHFCherry rats had the same body weight evolution as rats maintained on HFHF diet, with a higher fasting leptin level than HFHF rats (51.26 ± 4 ng/mL). HFHFCherry rats developed hyperglycaemia (1.45 ± 0.07 g/L), dyslipidaemia (TG: 131 ± 189 µM; Chol: 2.86 ± 0.29 mM; FFA: 354 ± 86 µM), glucose intolerance (area under the curve: 391 ± 25), and insulin resistance (HOMA-IR: 6.1 ± 0.95). Moreover, they developed C-peptidemia after 2 h of ipGTT at a faster rate than HFHF rats (*t0′*: 2423 ± 397 and *t60′*: 5841 ± 275 pmol/L) (Figs. 1, 2a–d).

However, the addition of cherries to the nutritional intervention where ND replaced the HFHF diet (HFHF/NDCherry) normalized glucose tolerance (area under the curve: 243.2 ± 10.32) and C-peptide levels during ipGTT (*t0′*: 1029 ± 118 and *t60′*: 3222 ± 368 pmol/L). Moreover, fasting glycaemia was under 1.26 g/L (1.24 ± 0.03 g/L) and cherry consumption decreased HOMA-IR (2.541 ± 0.29) (Fig. 1b–e). Plasmatic FFA levels were decreased (130 ± 11.7 µM) but not normalized to ND levels. However, hypercholesterolemia and hypertriglyceridemia were eliminated (1.52 ± 0.16 mM and 650 ± 167 µM, respectively) (Fig. 2b–d). The HFHF/NDCherry diet stopped weight gain until the end of the treatment and body weight, body mass index, abdominal circumference (Table 1, Fig. 1a) and leptinemia (20.8 ± 2.7 ng/mL) were normalized to ND levels (Fig. 2a).

### Beneficial effect of dietary intervention associated with cherry consumption on antioxidant capacity

HFHF rats had a twofold higher TBARS level in plasma than ND rats (62.80 ± 8.9 vs. 35 ± 1.1 µM MDA) (Fig. 2e). This oxidative stress complication was associated with an increase in superoxide dismutase (SOD) activity (71.43 ± 4.22 vs. 57.52 ± 5.83% of inhibition) and a decrease in catalase activity (0.022 ± 0.004 vs. 0.052 ± 0.03 µM) (data not shown). No difference in total antioxidant capacity was observed despite plasmatic oxidative stress (5.75 ± 0.17 vs. 5.75 ± 0.18 µM eq. Trolox) (Fig. 2f).

The HFHFCherry diet had no beneficial impact on plasmatic oxidative stress. In fact, HFHFCherry rats had the same levels of TBARS (52.21 ± 9.5 µM MDA) and SOD activity (78 ± 3.67% of inhibition) as HFHF rats, a great variability of catalase activity (neither significant vs. ND rats nor HFHF rats, 0.031 ± 0.014 µM) and also decreased total antioxidant capacity (4.36 ± 0.49 µM eq. Trolox) (Fig. 2e, f and data not shown).

However, the HFHF/NDCherry diet normalized plasmatic TBARS levels (33.41 ± 2.08 µM MDA) and catalase activity (0.043 ± 0.01 µM), had a higher SOD activity (81.87 ± 1.08% of inhibition) and increased total antioxidant capacity (6.62 ± 0.09 µM eq. Trolox) (Fig. 2e, f and data not shown).

### Cherry consumption emphasizes the beneficial effect of dietary intervention on hepatic oxidative stress and inflammation

**Fig. 3:**
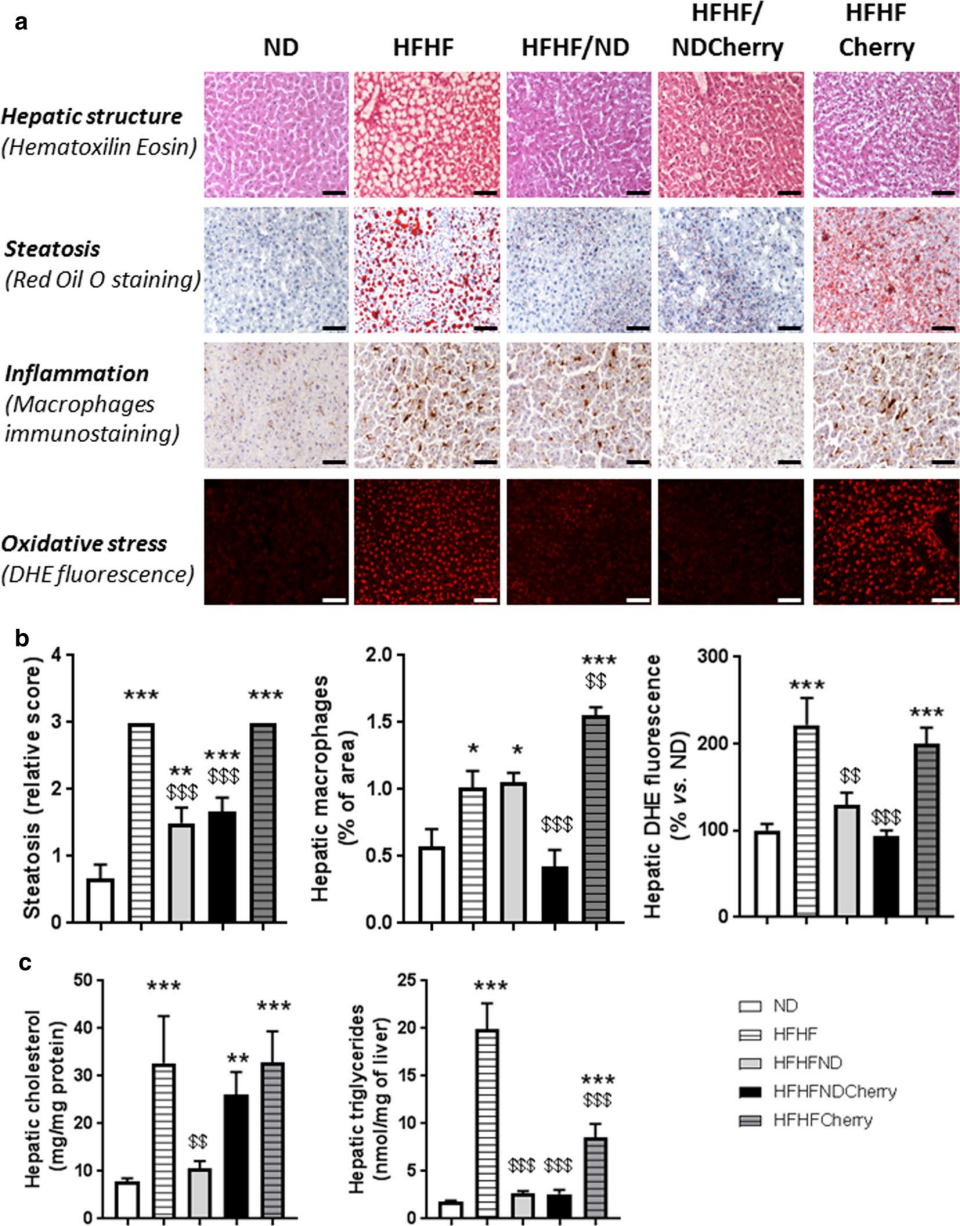
Impact of cherry consumption on hepatic complications 4 months into the experimental period. Impacts of cherry consumption on (**a**) hepatic structure assessed by eosin/hematoxylin coloration, steatosis by Oil-Red O, macrophages infiltration by immunohistochemistry and oxidative stress by dihydroethidine fluorescent probe (DHE) after 4 months of normal diet (ND), high fat high fructose (HFHF) diet, HFHF 2 months + ND 2 months (HFHF/ND), HFHF 2 months + ND with cherry supplementation 2 months (HFHF/NDCherry) and HFHF 2 months + HFHF with cherry supplementation 2 months groups (HFHFCherry). Bar scale = 100 µm; **b** relative score of steatosis, percentage of macrophages and DHE fluorescence quantifications; **c** hepatic cholesterol and triglycerides contents. All cumulative results are shown as the mean ± SEM of 6 different experiments. Asterisk represents significant results vs. ND; dollar vs. HFHF

Rats fed an HFHF diet showed a decrease in hepatic glycogen content (0.024 ± 0.003 vs. 0.042 ± 0.004 mg/mg of liver) and a maximal steatosis score after 4 months (3.0 ± 0 vs. 0.67 ± 0.21) (data not shown). Many cell nuclei were shifted from a location in the centre of the hepatocyte to the periphery, apparently due to interference from normal cell structures because of the presence of numerous large fat globules (Fig. 3a, b). These structural disorders were associated with a higher content of hepatic TG (19.9 ± 2.74 vs. 1.83 ± 0.08 nmol/mf of liver) and cholesterol (32.7 ± 9.87 vs. 7.82 ± 0.68 mg/mg protein) (Fig. 3c). These complications were associated with inflammation with macrophages infiltration (1.02 ± 0.12 vs. 0.57 ± 0.14% of area) and oxidative stress (222 ± 31.3 vs. 100 ± 7.7%) (Fig. 3a, b).

The HFHFCherry diet had no beneficial impact on plasmatic oxidative stress (Figs. 2f, 3). In fact, HFHFCherry rats had a great variability in glycogen content (neither significant vs. ND rats nor HFHF rats, 0.036 ± 0.005 mg/mg of liver), a maximal steatosis score (3.0 ± 0) with hepatic TG (8.52 ± 1.45 nmol/mf of liver) and cholesterol (32.85 ± 6.54 mg/mg protein) associated with a threefold macrophages infiltration (1.55 ± 0.1% of area) and oxidative stress (200 ± 18.6%).

However, the HFHF/NDCherry diet normalized macrophages infiltration (0.42 ± 0.13% of area), oxidative stress (93 ± 7.1%) and TG (2.58 ± 0.45 nmol/mf of liver) (Fig. 3a, b). HFHF/NDCherry rats presented an intermediate appearance and steatosis score (1.67 ± 0.2) associated with hepatic cholesterol (26 ± 4.76 mg/mg protein) and lower glycogen content than ND rats (0.0267 ± 0.0035 mg/mg of liver) (Fig. 3 and data not shown).

### Beneficial impact of cherry consumption on hepatic metabolic and oxidative pathways

**Fig. 4:**
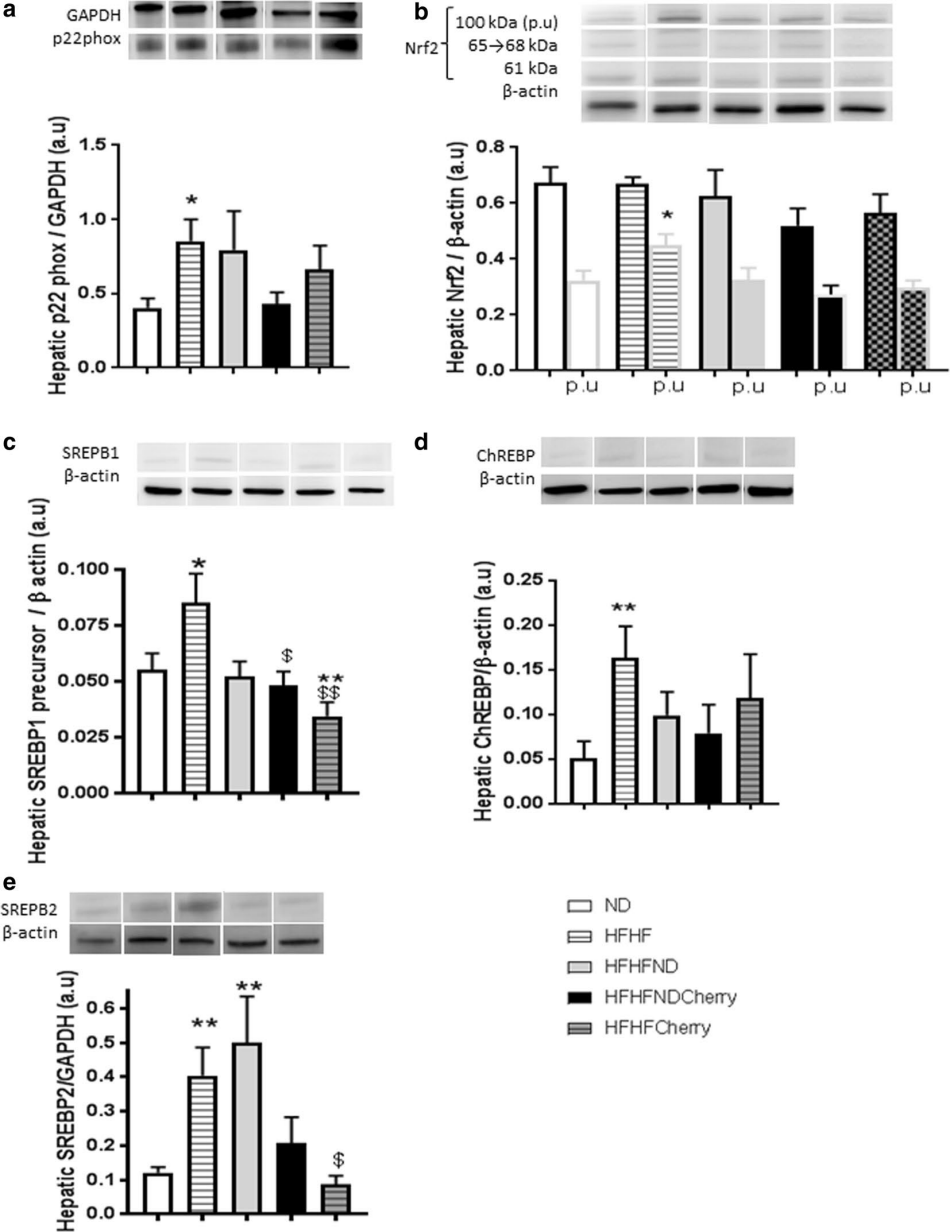
Impact of cherry consumption on liver 4 months into the experimental period. Impact of cherry consumption on oxidative parameters assessed by (**a**) the expression of p22phox and **b** the expression of Nrf2 (p.u: polyubiquitination). The impact on **c** triglycerides metabolism was assessed by SREBP1 and ChREBP expressions and **d** on cholesterol metabolism by the expression of SREBP2. Experiments show results after 4 months of normal diet (ND), high fat high fructose (HFHF) diet, HFHF 2 months + ND 2 months (HFHF/ND), HFHF 2 months + ND with cherry supplementation 2 months (HFHF/NDCherry) and HFHF 2 months + HFHF with cherry supplementation 2 months groups (HFHFCherry). All the results are shown as the mean ± SEM of 6 different experiments. Asterisk represent significant results vs. ND; dollar vs. HFHF

The HFHF diet induced hepatic increase of p22phox expression (0.85 ± 0.15 vs. 0.40 ± 0.06 a.u) (Fig. 4a), an NADPH oxidase (Nox) subunit and a major source of glucose-induced ROS production in liver [50, 53]. Moreover, Nrf2 polyubiquitination was increased 1.5-fold in HFHF rats in comparison to ND rats (0.45 ± 0.04 vs. 0.32 ± 0.03 a.u) (Fig. 4b), all leading to excessive hepatic ROS formation and oxidative stress as shown before. The HFHFCherry and HFHF/NDCherry diets preserved a physiological level of p22phox (respectively: 0.51 ± 0.10 and 0.66 ± 0.16 a.u) and Nrf2 expression and ubiquitination (respectively: 0.27 ± 0.03 and 0.30 ± 0.03 a.u) (Fig. 4a, b).

The HFHF diet increased the sterol regulatory element-binding protein-1c (SREBP-1c) (0.09 ± 0.01 vs. 0.06 ± 0.01 a.u), the carbohydrate-responsive element-binding protein (ChREBP) (0.16 ± 0.04 vs. 0.05 ± 0.02 a.u) and the master regulator of intracellular cholesterol homeostasis (SREBP2) (0.40 ± 0.08 vs. 0.12 ± 0.02 a.u), three major transcription factors implicated in liver lipogenesis [54, 55]. The HFHFCherry diet decreased SREBP1 (0.035 ± 0.01 a.u), tended to increase ChREBP (0.12 ± 0.05 a.u; P = 0.08) and normalized SREBP2 (0.09 ± 0.02 a.u) expressions in comparison to the ND whereas the HFHF/NDCherry diet preserved a physiological level of SREBP-1c (0.05 ± 0.01 a.u), ChREBP (0.08 ± 0.03 a.u) and SREBP2 (0.21 ± 0.08 a.u) (Fig. 4c–e).

### Dietary intervention associated with cherry supplementation maintained vascular homeostasis

**Fig. 5:**
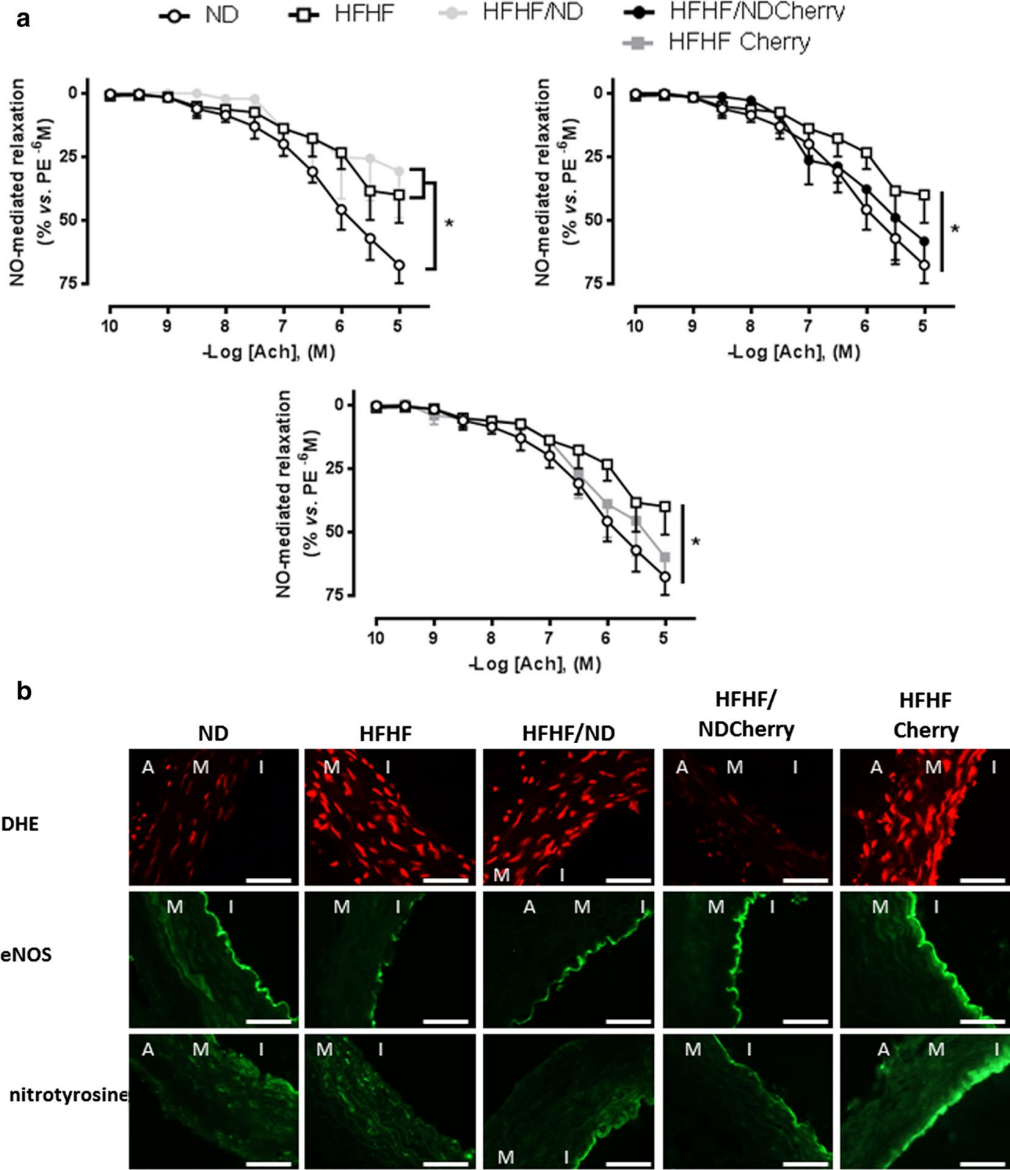
Impact of cherry consumption on vascular complications 4 months into the experimental period. Impacts of cherry consumption on **a** vascular function represented by NO-mediated relaxation induced by acetylcholine (Ach) on phenylephrine (PE) pre-contracted mesenteric artery and **b** characterization of endothelial dysfunction through the presence of reactive oxygen species by dihydroethidine fluorescent probe (DHE), the localization and expression of eNOS and the formation of nitrotyrosine after 4 months of normal diet (ND), high fat high fructose (HFHF) diet, HFHF 2 months + ND 2 months (HFHF/ND), HFHF 2 months + ND with cherry supplementation 2 months (HFHF/NDCherry) and HFHF 2 months + HFHF with cherry supplementation 2 months groups (HFHFCherry). *A* adventice, *M* media, *I* intima; bar scale = 50 µm. All the results are shown as the mean ± SEM of 6 different experiments. Asterisk represents significant results vs. ND

HFHF rats had a decrease of NO-mediated relaxation in the mesenteric artery associated with oxidative stress and decreased expression of eNOS in the endothelium of the vessel (Fig. 5). In fact, acetylcholine, which induced relaxation via a calcium dependent pathway [52], caused NO-mediated concentration-dependent relaxations in mesenteric artery rings in ND rats (at 10^−5^ M, 67 ± 7%) associated with eNOS expression in the vessel (100 ± 10.73%). However, blunted NO-mediated relaxation was observed in HFHF rats (at 10^−5^ M, 40 ± 11%), associated with a twofold decrease in eNOS (53.35 ± 7.17%) and a threefold ROS formation in all the vasculature, as observed with DHE fluorescence (339 ± 56.3% vs. 100 ± 7.14%.). Decrease in NO was not correlated with peroxintrite formation (association of ROS and NO) as observed by physiological nitrotyrosine fluorescence levels (113.5 ± 14.97 vs. 100 ± 8.32%) (Fig. 5b).

HFHFCherry and HFHF/NDCherry rats experienced an intermediate NO-mediated relaxation (respectively at 10^−5^ M, 59.77 ± 16.22%; 58.09 ± 18.80%) over 2 months, similar to ND and HFHF rats. However, HFHFCherry rats presented excessive ROS (338 ± 44.1%) in all the vasculature associated with endothelial peroxinitrite (194.5 ± 26.45%) and a compensatory increase of eNOS expression (156.3 ± 26.3%). In contrast, the HFHF/NDCherry diet prevented ROS and nitrotyrosine formation (respectively: 98.3 ± 18.6%; 129.46 ± 14.43%) and presented a physiological level of eNOS (108.3 ± 11.7%) (Fig. 5).

### No impact of dietary intervention associated with cherry supplementation on pancreas oxidative stress

The HFHF diet induced an increase in ROS in the entire pancreas (212.30 ± 25.2% vs. 100 ± 12%). The HFHFCherry and HFHF/NDCherry diets had no effect on pancreatic oxidative stress because of the formation of ROS in the pancreas, including islets, as observed in HFHFCherry rats (172.6 ± 13.3%) and HFHF/NDCherry rats (234 ± 32.7%) (Additional file 2: Figure S1).

**Fig. 6:**
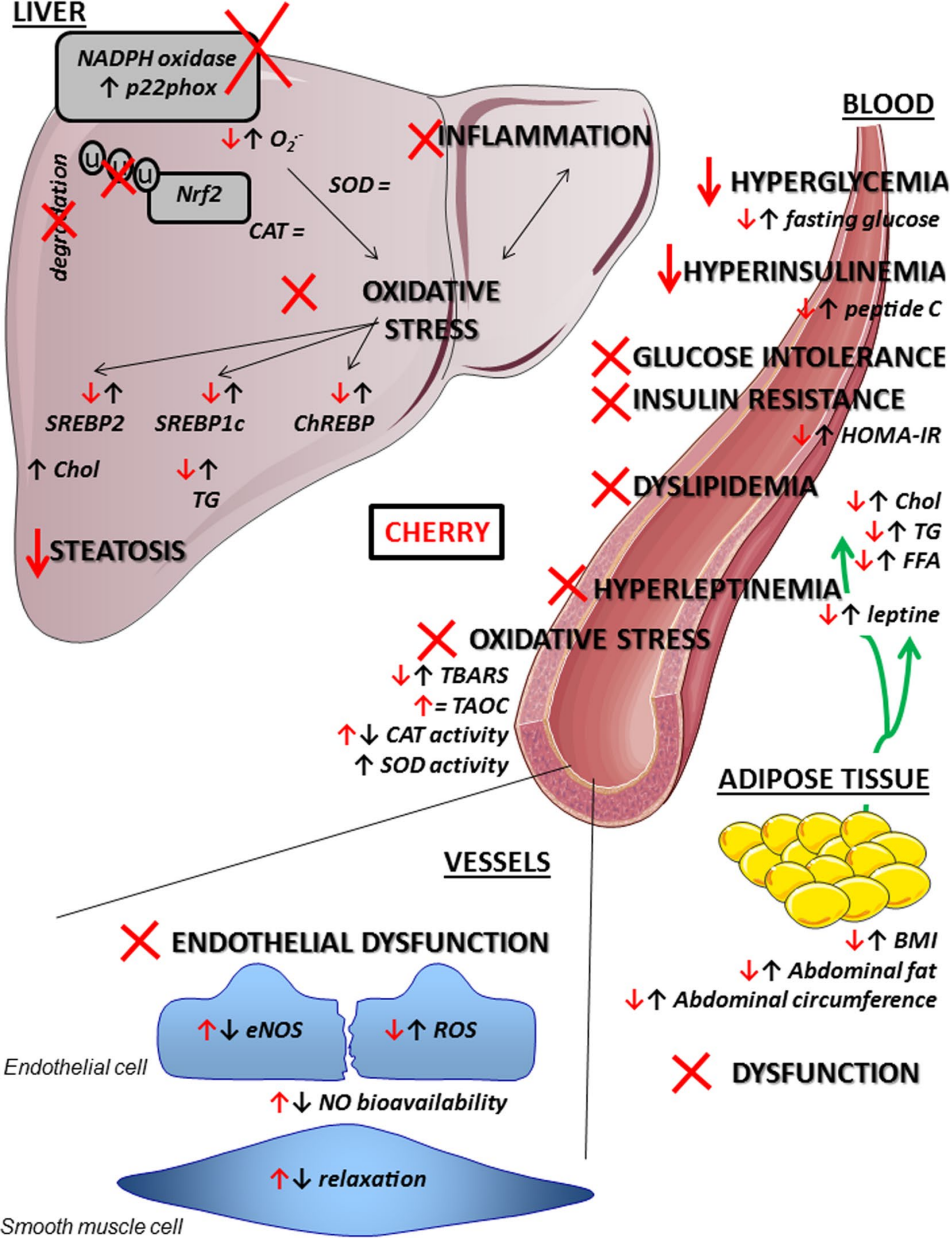
Disorders observed in HFHF rats and beneficial impacts of cherry consumption in addition to nutritional intervention. Cherry consumption associated with nutritional strategy has multiple beneficial effects against complications induced by T2D. In blood, cherry consumption decreased fasting glucose, C-peptide and HOMA-IR leading to reduced hyperglycaemia and hyperinsulinemia, assuring physiological glucose and insulin tolerance. Cherry consumption decreased adipose tissue dysfunction and thus decreasing weight gain, body mass index (BMI) and abdominal circumference. Cherry consumption normalized adipokines secretion [leptine, cholesterol (Chol), triglycerides (TG) and free fatty acids (FFA)] and then eliminated dyslipidemia and hyperinsulinemia. Cherry consumption decreased systemic oxidative stress and thiobarbituric acid reactive substances (TBARS) complications by increasing catalase (CAT) activity and total antioxidant capacity (TAOC). All these systemic beneficial effects of cherry consumption led to suppressed endothelial dysfunction. Nitric oxide (NO) bioavailability was increased in accordance with the decrease of reactive oxygen species (ROS) production and the increase of the endothelial NO synthase (eNOS) leading to physiological relaxation of the vessel. Cherry consumption in the liver decreased p22phox, a subunit of the nicotinamide adenine dinucleotide phosphate oxidase (NADPH oxidase) and thus decreased superoxide anion (O_2_^−^) formation. In addition, cherry consumption inhibited the ubiquitin-degradation of the nuclear factor erythroid-2 related factor 2 (Nrf2), all leading to eliminated oxidative stress and linked inflammation. Additionally, cherry consumption decreased carbohydrate-responsive element-binding protein (ChREBP) and sterol regulatory element-binding proteins (SREBP-1 and -2), then decreased TG accumulation and steatosis. SOD: superoxide dismutase, upward arrow increase, downward pointing arrow decrease, X: suppress. Black information: effect of diabetes; red information: effect of cherry consumption

Figure 6 presents all disorders observed in HFHF rats and highlights the beneficial impacts of cherry consumption and nutritional intervention on blood, vessels, and liver.

## Discussion

In our study, we have demonstrated that cherry consumption decreased the risk of developing diabetic disorders by reducing fat accumulation, body weight and lipid concentrations and improving glucose and insulin regulation, enhancing metabolic and oxidative balance in plasma. Moreover, it helped in maintaining an anti-oxidant and anti-inflammatory state leading to decreased vascular and hepatic complications.

### Cherry consumption improves plasmatic oxidative and metabolic disorders associated to diabetes

Firstly, we have demonstrated that cherry consumption decreased oxidative stress in plasma. While diabetes induces a decrease in catalase activity and TBARS production without any modulation of TAOC, cherry consumption was able to increase catalase activity and TAOC leading to a decrease in TBARS complications. Accordingly, studies in healthy human subjects reported that cherry consumption increases plasma TAOC [43, 56]. Traustadottir et al., in a double blind placebo-controlled crossover design in older adults, showed that consumption of tart (sour) cherry juice improves antioxidant defences by increasing the capacity to constrain an oxidative challenge and reducing oxidative damage to nucleic acids [57]. Moreover, cyanidin-3-rutinoside, present in our cherry extract [35], displays a wide range of biological activities, including antioxidant and anti-inflammatory [58]. Recently, an in vitro study confirmed the beneficial impact of cyaniding-3-rutinoside on oxidative stress damage and the inhibition of TBARS formation in bovine serum albumin [59]. Recent epidemiological studies highlighted that people with acatalasemia develop T2D [60] and Hait et al. have demonstrated that catalase deletion promotes obesity associated with the impairment of glucose tolerance and insulin sensitivity, increased plasmatic TGs and induced steatosis and inflammation in the liver of Cat^−/−^ mice [61]. Our results are in accordance with these data and suggested that cherry consumption could prevent alterations in lipids mobilization and utilization thought their beneficial effect on catalase, thus avoiding excess circulating lipids. Therefore, all these data highlight not only the improvement of systemic oxidative balance with cherry consumption but also an improvement of lipids profiles.

Besides the beneficial impact on redox homeostasis, cherry consumption normalized glucose tolerance, insulin resistance, dyslipidaemia, hyperleptinemia, decreased hyperglycaemia, and hyperinsulinemia. These beneficial impacts of cherry consumption could be closely linked to its ability to decrease obesity and inhibit adipocyte dysfunction, two disorders strongly associated with the development of insulin resistance, cell impairment and T2D [62, 63]. In fact, anthocyanins are considered modulators of adipose tissue metabolism which improve adipocytes dysfunction and adipocytokines secretion in insulin resistance, increase β-oxidation and decrease fat accumulation on adipocytes [64]. Hypertrophic adipocytes, which release rather than store FFAs, are linked to insulin resistance [65], but were decreased by cherry consumption, which could explain the normalization of glucose tolerance, insulin sensitivity, leptinemia and dyslipidaemia. All these beneficial effects were observed only when cherry consumption was associated with ND and not with the HFHF diet. Then, our results suggest that cherry consumption may cause lipid trafficking away from the abdomen and hence reduce the associated complications, mainly NASH and cardiovascular dysfunction. These findings are in accordance with some in vivo studies which demonstrated that cherries and their bioactive food components decrease body weight and abdominal fat [66], blood lipids [67, 68] and fasting blood glucose [64, 66]. More precisely, Cherian et al. demonstrated that a single dose of anthocyanins decreases fasting glycaemia by 19% and improves glucose tolerance by 29% in moderately-diabetic rats. Moreover, 4 weeks of treatment dropped the pre-treatments levels of fasting blood glucose by 50% and increased glucose tolerance by 41% [69]. Similar results were observed in high fat diet-rats with 5-caffeoylquinic acid [70], one of the compounds in Regina cherries [35]. Another therapeutic approach to treat diabetes is to delay the absorption of glucose via inhibition of enzymes, such as α-glucosidase, in the digestive organs. It has been confirmed that α-glucosidase activity in vitro can be inhibited by berry extracts rich in polyphenols [71] and by cyanidin-3-rutinoside [72], a derivate of anthocyanin present in the cherry extract used in our study [35]. All these data highlight the fact that bioactive food compounds found in cherries are responsible for an improvement in systemic metabolic balance.

### Cherry promotes NO bioavailability and assures vascular function

Keeping a state of oxidative, carbohydrate and lipid homeostasis is essential to ensuring vascular function. In fact, the endothelium, the internal layer of vessels, is in constant interaction with the blood, subjected to mechanical and chemical stresses, and plays a pivotal role in vascular homeostasis. A better understanding of the cellular basis of the pathophysiological processes and better strategies to treat damage are clearly an important goal because diabetes-associated vascular complications are responsible for 75% of the deaths associated with diabetes [73]. We have demonstrated that HFHF induced diabetes is associated with a decrease of relaxation in the mesenteric artery involving a decrease of eNOS expression and, thus, blunted NO-mediated relaxation, in addition to ROS formation. This endothelial dysfunction has been associated in several regions of the vasculature in animals and humans with T2D due to defects in NO-derived vasodilation [74–77]. A number of studies have suggested that ROS play an important role in the pathogenesis of diabetic vasculopathy, affecting both the macro- and the microvascular systems [63, 78]. All metabolic disorders observed in blood in our model could be linked to disturbance of vessels [76]: high cholesterol and FFA, obesity and visceral fat distribution, insulin resistance, impaired fasting glucose and glycaemic fluctuations [7, 79, 80]. They have also been associated with ROS formation and exacerbated oxidative stress [7, 12, 63, 81]. However, as shown before in our study, cherry consumption was able to assure blood homeostasis, leading to decreases in oxidative stress in vessel vasculature, to increases eNOS expression and thus to assure good NO-bioavailability and relaxation in HFHF/NDCherry rats. Much of research suggests, in fact, the cardio-protective effects of cherry consumption and anthocyanins appear to have some vasoprotective effects in humans [82]. Endothelial cells from bovine arteries exposed for several hours to cyanidins increased NO output and reduced local oxidative stress [83], decreased inflammation and indirectly reduced the risk of atherosclerosis plaque formation [84]. In fact, polyphenols, such as citrus flavonoids or isoflavones from red clover, could increase flow mediated dilatation and improve vascular function after 3–4 weeks in patients with metabolic syndrome [85, 86] and anthocyanins, including cyanidin-3-rutinoside as shown in our cherry composition, have potent platelet-inhibitory properties and are considered inhibitors of platelet cell signalling and thrombus formation [87]. Moreover, cherry consumption inhibited free radical formation which could prevent the onset and development of long-term diabetic complications [88]. This vascular protection of cherry consumption against oxidative stress has been closely correlated to the improvement of metabolic and lipidic profiles in blood. In fact, the HFHFCherry diet has no beneficial impact on these plasmatic parameters and thus exhibited ROS in all the vasculature. However, in this nutraceutical strategy, eNOS was highly increased by cherry supplementation to counteract ROS and peroxinitrite formation and thus to assure NO bioavailability and relaxation in vessels, as shown in HFHF/Cherry-rats. Changes in endothelial function hasten the development of micro- and macroangiopathy and thus target the cellular basis of endothelial dysfunction and promote NO bioavailability. Cherry supplementation, in addition to lifestyle measures, should provide benefits to the overall therapeutic management of diabetes.

### Cherry promotes NO bioavailability leading to optimal metabolic function

In addition to its vascular beneficial effect, NO derived from eNOS appears to have both antiobesogenic and insulin-sensitizing properties. These effects, discovered in recent years, are due to its ability to increase fat oxidation in peripheral tissues, such as liver and adipose tissue, to decrease lipid synthesis in the liver, increase insulin and glucose transports to key peripheral tissues and to regulate gluconeogenesis [89]. These metabolic effects of NO bioavailability could explain in part the beneficial impact of cherry consumption on the liver. In fact, massive hepatic lipid accumulation observed in HFHF rats in our study and in T2D patients [90] was eliminated in HFHF/NDCherry rats. Our findings are in line with an earlier study in HFD-mice and consumption of a mixture of pure anthocyanins [66]. Seymour et al. also reported a decrease in hyperlipidemia, hyperinsulinemia, fatty liver and hepatic steatosis [68] with 90-day administration of sour cherry. Moreover, supplementation of the HFD-rats with 5-caffeoylquinic acid, one of our cherry compounds [35], reduced macrophage infiltration and steatosis [70] via PPARγ and NFκB signaling pathways. Today, these pathways, such as antioxidants inhibiting NADPH oxidase, receive a lot of attention in the treatment of atrial fibrillation [91]. So, our results, in addition to others, highlighted the anti-steatosic effect of cherry consumption.

### Cherry improved NASH associated to diabetes: anti-FFAs and anti-oxidant beneficial effects

While many mechanisms can explain the improvement of hepatic steatosis and NASH complication, normalization of FFAs and oxidative stress by cherry consumption seem to be involved in our study. Li et al. demonstrated that FFAs cause hepatic insulin resistance, resulting in overproduction of glucose and hyperglycemia, and initiate inflammatory processes in the liver, thus resulting in the development of steatohepatitis [65]. Additionally, Pereira et al. linked FFAs to NADPH oxidase and oxidative stress in impaired hepatic insulin signalling [92]. Numerous disorders stimulate NADPH oxidase activity: elevated glucose, hyperinsulinemia, lipids, and cytokines [93]. As shown before, only cherry consumption in association with lifestyle measures (ND) normalized them in plasma and in the liver cherry consumption decreased HFHF-induced p22phox expression (NADPH oxidase subunits), ROS formation and NASH (steatosis plus inflammation). Previous studies demonstrated that anthocyanins reduce ROS generation in human HepG2 cells exposed to a high glucose environment [94], increase activity of the antioxidant enzymes SOD (liver, blood) and Gpx (liver) and decrease lipid peroxidation [95]. One of the major defence systems against stress-related injury is the Nrf2 system [10, 96] which activates the antioxidant response elements. Activation of Nrf2 by a number of polyphenols increases expression of phase II detoxifying enzymes and antioxidant enzymes [97, 98], which can directly act to eliminate free radicals and oxidative damaged molecules. Recently, Nrf2 was highlighted as a real target against diabetic nephropathy [10]. Our results showed that the HFHF diet increased Nrf2 degradation in the liver and was clearly associated with the presence of oxidative stress. But cherry supplementation avoided this degradation, in addition to decreasing p22phox expression and ROS formation.

### Cherry improved NASH associated to diabetes: involvement of hepatic pathways

Degradation of Nrf2 was also implicated in the development of NASH [99, 100] because of its role in lipid catabolism [101]. SREBPs (-1 and -2) and ChREBP regulate the gene expression of enzymes involved in lipogenesis and cholesterol synthesis [54, 102]. Our present study demonstrated that the HFHF diet induced an abnormal expression profile of these hepatic lipogenic transcription factors, increasing de novo lipogenesis due to hyperglycemia and hyperinsulinemia [55, 103] as observed in our model. However, cherry consumption was able to normalize them, decrease hepatic TGs and steatosis and thus decrease plasmatic dyslipidemia and hyperglycemia. Polyphenols, including anthocyanins, significantly reduce tissue lipid accumulation and the activity of enzymes that promote fat storage [104], and also lower body weight, fat mass and TGs through enhancing energy expenditure, fat utilization and modulating glucose hemostasis [105]. Little data are available on the in vivo effect of anthocyanins or cherry consumption on these hepatic targets. Anthocyanin from mulberry extract decreases SREBP1c and SREBP2 on human hepatocyte (HepG2) cultured with high fatty acid, suppressing fatty acid synthesis and enhancing fatty acid oxidation, all contributing to amelioration of lipid accumulation [106]. Additionally anthocyanins from purple sweet potato decrease SREBP1c in the same in vitro model but also in vivo in HDF-mice [107]. Musso et al. reviewed cellular mechanisms of cholesterol toxicity involved in liver injury and NASH and highlighted the therapeutic impact of anthocyanin through a decrease SREBP2 and lipogenesis [104]. So, all these data highlight the fact that the cherry is responsible for an improvement of hepatic complications associated with a decrease of oxidative stress and inflammation.

### Antioxidant therapy: the practical implications

Our recent data showed that the consumption of cherry without any metabolic disorders, so on healthy rats, leads to opposite effects. For example, hepatic p22phox expression was increased, leading to oxidative stress and associated to hepatic dysfunction (unpublished data). These results highlight the difficulty to work with antioxidant compounds, which could be pro-oxidant sometime. We worked also with cherry which contained fructose as the major source of sugar; future study could be done with free-sugar cherry extract, but only for the purpose of a consumption of modified food and not the promotion of the consumption of natural healthy food. Identifying specific polyphenolic compound(s) in cherry extract leading to the beneficial effect could be also a strategy, but as highlighted by Snyder et al. [108] ‘a challenge for future research is not only to describe the improvements produced by the intake of specific healthful foods or phytochemicals, but also to determine what beneficial synergies may be produced by consuming complementary healthy foods containing a variety of bioactive compounds, acting on multiple and molecular-level regulatory pathways’. Several studies in animal models and human subjects have demonstrated that phenols are bioavailable and exert a protective role against oxidative stress and free radical damage [30, 40, 82]. Moreover epidemiological studies suggest that consumption of fruits, vegetables and plants [30] may be associated with a reduced risk of diabetes or have a protective effect [109]. Recently, Pickering et al. [10] clearly reviewed the feasibility of emerging new therapies to combat oxidative stress and inflammation in the diabetic milieu. The use of therapy like cherry brings a real asset thanks to its broad-spectrum effects on: (1) the regulation of carbohydrate and lipid metabolisms, (2) the attenuation of oxidative damage and scavenging of free radicals, (3) the improvement of endothelial function and vascular tone through the enhancement vasodilation factor production, and (4) the decrease of NASH with macrophages and ROS inhibition. All the bibliography available today on the subject brings hope on using antioxidants in future hepatitis and antidiabetic therapeutics.

## Conclusion

Medical nutrition therapy is recommended for all patients with T2D and, along with activity, is a cornerstone of treatment. Nevertheless, a recent widely discussed study failed to achieve a reduction of cardiovascular events in overweight or obese adults with T2D after a 10-year intense lifestyle intervention, despite improvements in body weight, physical fitness, and metabolic markers [110]. Despite the presence of known antidiabetic medicine in the pharmaceutical market, diabetes and its related complications continue to be a major medical problem. In recent years, we have come to understand diabetes-associated vascular and hepatic complications as clearly linked disorders. Interconnected failures include adipose tissue, blood vessels, endothelial function and liver, which is why new therapies need to act on several points. Consumption of bioactive food, such as cherries, provides a unique combination of phytonutrients in one package that work together to deliver health benefits. Their pleiotropic effects could be an interesting target in order to optimize management of long-term diabetic complications.

## Additional files

**Additional file 1: Table S1.** Food composition and food and beverage consumption.

**Additional file 2: Figure S1.** Impact of cherry consumption on pancreatic oxidative stress 4 months into the experimental period. Oxidative stress is assessed by dihydroethidine fluorescent probe (DHE) after 4 months of normal diet (ND), high fat high fructose (HFHF) diet, HFHF 2 months + ND 2 months (HFHF/ND), HFHF 2 months + ND with cherry supplementation 2 months (HFHF/NDCherry) and HFHF 2 months + HFHF with cherry supplementation 2 months groups (HFHFCherry). Bar scale = 100. All the results are shown as the mean ± SEM of 6 different experiments. Asterisk represents significant results vs. ND; $ vs. HFHF.

## Abbreviations

ABTS: 2,2′-azino-bis-(3-ethylbenzthiazoline-6-sulfonic acid)
Chol: cholesterol
ChREBP: carbohydrate-responsive element-binding protein
DHE: dihydroethidine
eNOS: endothelial nitric oxide synthase
FFA: free fatty acids
HFHF: high fat high fructose
HFHFCherry: rats who received 2 months of HFHF diet and shifted to 2 months of HFHF diet enriched with cherries
HFHF/ND: rats who received 2 months of HFHF diet and shifted to 2 months of ND
HFHF/NDCherry: rats who received 2 months of HFHF diet and shifted to 2 months of ND enriched with cherries
HOMA2-IR: homeostatic model assessment indexes of insulin resistance
HPLC: high performance liquid chromatography
ipGTT: intraperitoneal glucose tolerance test
NADPH oxidase: nicotinamide adenine dinucleotide phosphate oxidase
NAFLD: non-alcoholic fatty liver disease
NASH: non-alcoholic steatohepatitis
ND: normal diet
NO: nitric oxide
Nrf2: nuclear factor erythroid-2 related factor 2
p22phox: a subunit of NADPH oxidase
ROS: reactive oxygen species
SOD: superoxide dismutase
SREBP(-1, -2): sterol regulatory element-binding proteins
TAOC: total antioxidant capacity
TBARS: thiobarbituric acid reactive substances
TG: triglycerides
T2D: type 2 diabetes
