# Supplementary material for: Beneficial effects of cherry consumption as a dietary intervention for metabolic, hepatic and vascular complications in type 2 diabetic rats
Source: Cardiovasc Diabetol. 2018 Jul 20;17:104. doi: 10.1186/s12933-018-0744-6 (PMC6054718; doi:10.1186/s12933-018-0744-6)
Supplement: Supplementary file 1 — Additional file 1: Table S1. Food composition and food and beverage consumption. [file 12933_2018_744_MOESM1_ESM.pptx]

## Slide 1
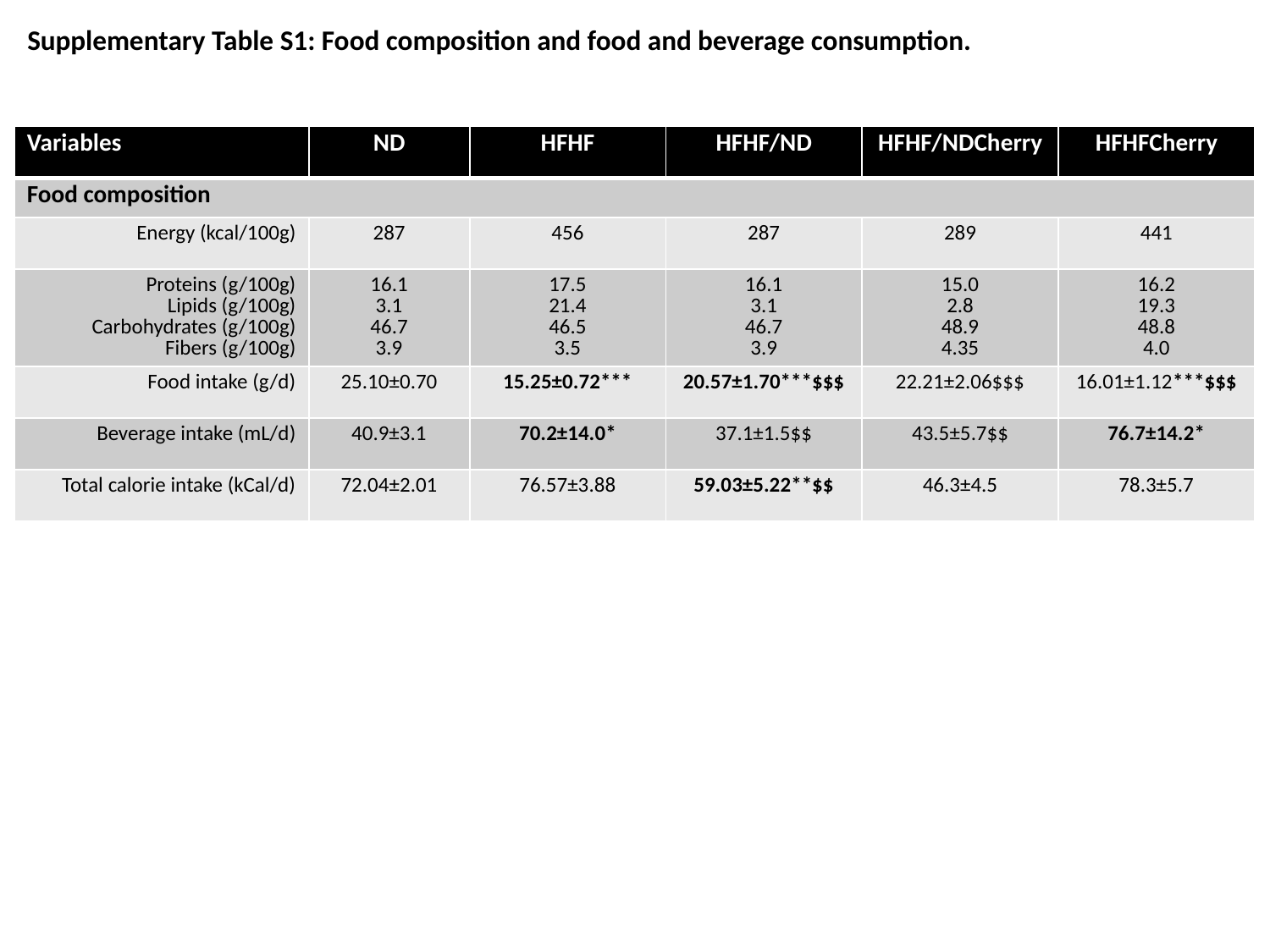

Supplementary Table S1: Food composition and food and beverage consumption.
| Variables | ND | HFHF | HFHF/ND | HFHF/NDCherry | HFHFCherry |
| --- | --- | --- | --- | --- | --- |
| Food composition | | | | | |
| Energy (kcal/100g) | 287 | 456 | 287 | 289 | 441 |
| Proteins (g/100g) Lipids (g/100g) Carbohydrates (g/100g) Fibers (g/100g) | 16.1 3.1 46.7 3.9 | 17.5 21.4 46.5 3.5 | 16.1 3.1 46.7 3.9 | 15.0 2.8 48.9 4.35 | 16.2 19.3 48.8 4.0 |
| Food intake (g/d) | 25.10±0.70 | 15.25±0.72\*\*\* | 20.57±1.70\*\*\*$$$ | 22.21±2.06$$$ | 16.01±1.12\*\*\*$$$ |
| Beverage intake (mL/d) | 40.9±3.1 | 70.2±14.0\* | 37.1±1.5$$ | 43.5±5.7$$ | 76.7±14.2\* |
| Total calorie intake (kCal/d) | 72.04±2.01 | 76.57±3.88 | 59.03±5.22\*\*$$ | 46.3±4.5 | 78.3±5.7 |
