# Supplementary figures and images for: Beneficial effects of cherry consumption as a dietary intervention for metabolic, hepatic and vascular complications in type 2 diabetic rats
Source: Cardiovasc Diabetol. 2018 Jul 20;17:104. doi: 10.1186/s12933-018-0744-6 (PMC6054718; doi:10.1186/s12933-018-0744-6)

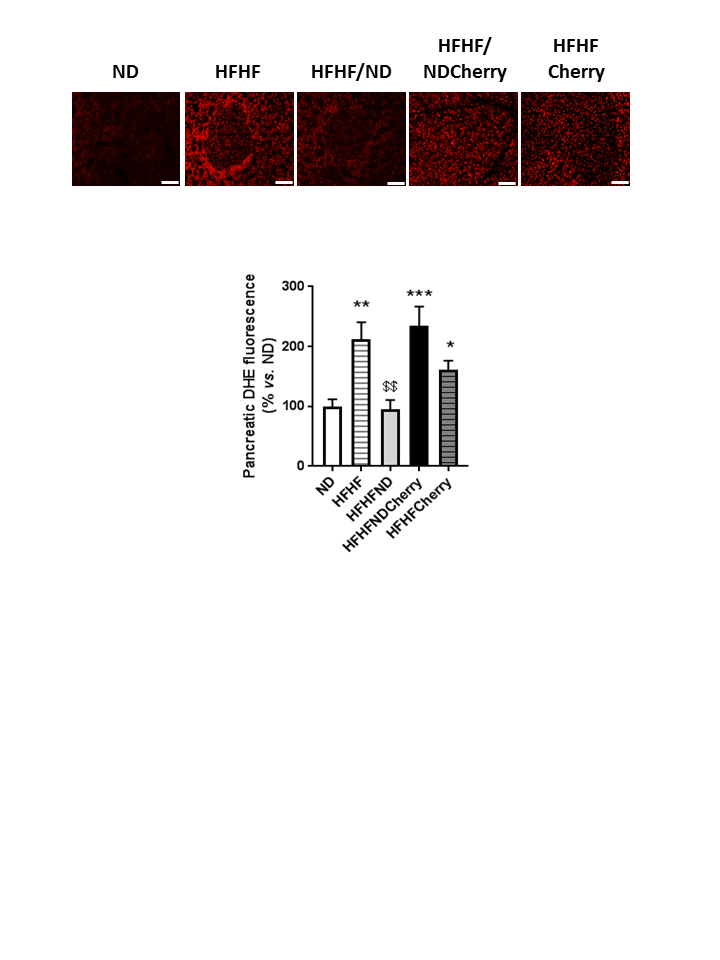

Supplement: Supplementary file 2 — Additional file 2: Figure S1. Impact of cherry consumption on pancreatic oxidative stress 4 months into the experimental period. Oxidative stress is assessed by dihydroethidine fluorescent probe (DHE) after 4 months of normal diet (ND), high fat high fructose (HFHF) diet, HFHF 2 months + ND 2 months (HFHF/ND), HFHF 2 months + ND with cherry supplementation 2 months (HFHF/NDCherry) and HFHF 2 months + HFHF with cherry supplementation 2 months groups (HFHFCherry). Bar scale = 100. All the results are shown as the mean ± SEM of 6 different experiments. Asterisk represents significant results vs. ND; $ vs. HFHF. [file 12933_2018_744_MOESM2_ESM.tif]
